# Numerical Evaluation of Seismic Performance of RC Columns Strengthened with Stiff-Type Polyurea

**DOI:** 10.3390/ma18081839

**Published:** 2025-04-17

**Authors:** Tae-Hee Lee, Soo-Ho Han, Jang-Ho Jay Kim

**Affiliations:** 1 School of Civil and Environmental Engineering, Yonsei University, 50, Yonsei-ro, Seodaemun-gu, Seoul 03722, Republic of Korea; saintlth@yonsei.ac.kr; 2 Foresys Co., Ltd., 26, Seongmisan-ro 1-gil, Mapo-gu, Seoul 03971, Republic of Korea; sooho.han@frss.co.kr

**Keywords:** RC column, glass fiber-reinforced polyurea (GFPU), stiff-type polyurea (STPU), FE modeling, seismic reinforcement

## Abstract

After developing the experimental database of RC column specimens retrofitted with stiff-type polyurea (STPU), this study implemented STPU in finite-element (FE) modeling. The numerical analysis aimed to evaluate seismic performance factors by establishing a structural analysis model based on the experimental data. The model was calibrated and validated against experimental results, showing consistency in maximum displacement and strain within acceptable deviations. The key findings indicate that the dissipation energy and crack propagation were significantly reduced in reinforced specimens compared to unreinforced ones, demonstrating the effectiveness of STPU and glass fiber-reinforced polyurea (GFPU). The FE model further confirmed that circular specimens exhibited superior reinforcement effects compared to rectangular specimens due to their continuous surface geometry. These results enhance the understanding of STPU’s seismic reinforcement capabilities and provide a foundation for its practical application. The study results are discussed in detail in the paper.

## 1. Introduction

Recently, earthquakes with a magnitude of 2.0 or more on the Richter scale have occurred in South Korea, including the Gyeong-Ju earthquake (12 September 2016) and Po-Hang earthquake, with a magnitude of 5.4 on the Richter scale (15 November 2017), the largest observed earthquakes in South Korea [1,2]. Based on the Earthquake and Disaster Countermeasures Act enacted in 2009, the 2017 earthquake-resistance design standards were revised in South Korea, and their application to all structures was announced. However, the social infrastructure structures built before the enforcement decree were still vulnerable to seismic load. The necessity for the maintenance and reinforcement of earthquake-vulnerable RC structures is increasing [3]. The seismic resistance effect of newly developed stiff-type polyurea (STPU) for the maintenance and reinforcement of RC structures was verified through experiments [4].

Research on the reinforcement performance evaluation of structures enhanced with Polyurea has been conducted through experiments and FEM simulations. Samiee et al. (2013) examined the explosive load resistance of a steel plate reinforced with PU using the LS-DYNA explicit finite-element analysis program [5]. When the coating thickness of PU exceeded 2 mm, better reinforcing effects were achieved when PU reinforcement was applied to the other side of a steel plate subjected to explosive loads. Parniani and Toutanji (2015) performed a flexural load test and evaluated the fatigue performance of PU-coated RC beams. In the flexural load test, the PU 5 mm-coated RC beam (P-B-M-2), PU 2.5 mm-coated RC beam (P-B-M-1), and the uncoated RC beam (C-M-B) demonstrated superior performance in that order, validating the reinforcing effect of PU for flexural loads. The difference in fatigue performance between PU with thicknesses of 2.5 mm and 5 mm was not significant, indicating that both thicknesses have almost identical behavior under fatigue load conditions [6]. Wang et al. (2017) evaluated the blast resistance of masonry walls reinforced with PU. PU reinforcement enhanced blast resistance by 4.5–11 times [7]. Song et al. (2020) reinforced RC columns using glass fiber-reinforced polyurea (GFPU) and measured the shear strength under quasistatic cyclic loading. The shear strength of the GFPU-reinforced columns was approximately 8.7% greater than that of the unreinforced RC columns [8]. In addition to experimental and finite-element investigations of polyurea-retrofitted RC columns, recent research has focused on advancing analytical approaches to better characterize the seismic behavior of RC structures. Kuria and Brassai (2023) presented a comprehensive review of pushover analysis techniques developed over the past three decades, highlighting their evolution and applicability in seismic engineering for assessing structural performance [9]. In terms of seismic damage modeling, Xu et al. (2024) developed probabilistic curvature limit states for corroded circular RC bridge columns, providing insight into the effects of material deterioration and cyclic flexural failure [10]. Furthermore, Asghar et al. (2022) applied gene-expression programming to predict the lateral load capacity of rectangular RC columns, demonstrating a robust approach to parameter calibration using experimental data. These recent developments enhance the theoretical foundation and practical tools available for simulating and assessing the seismic performance of RC columns [11].

In this paper, FE model is constructed for RC columns reinforced with STPU using the experimental data from Lee et al. (2022) paper [4]. Building FE modeling in dynamic inelastic analysis is an important tool for the evaluation of the performance of RC structures under strong earthquakes. FE modeling and FE analysis allow us to reduce the cost associated with experiments and enables the identification of other factors that may not have been observable during experimentation [12]. In this paper, a 3D FE model was constructed using ABAQUS, and the calibration of the FE model was conducted using the experimental data from Lee et al. (2022) paper [4]. With the calibrated model, the extent of damage for each specimen was assessed to compare and analyze the reinforcement performance under seismic load. The numerical results demonstrate excellent agreement with experimental data and confirm the effectiveness of the proposed strengthening method in reducing damage and enhancing energy-dissipation capacity.

Lee et al. (2022) [4] presented the seismic performance evaluation of RC column retrofitted by STPU through pseudo-dynamic and shaking table tests. In this paper, the seismic reinforcement performance of STPU was evaluated by developing a 3D FE model based on the experimental results. All the notations from the Lee et al. (2022) [4] paper will be retained.

The scope of the study is to develop a simulation model for PU-strengthened RC columns for enhancement of seismic resistance. Within the model, a hybrid strengthening of GFRP with PU is included. The importance of developing and calibrating the model is to be able to perform various design parametric study for real-scale PU-strengthened RC columns. Based on the comparison of the experimental and simulation results, the model is verified for the design usage purpose in the future.

## 2. FE Modeling

In order to evaluate the strengthening effect and stiffness enhancement from the application of STPU, the Implicit ABAQUS (2020) commercial FE program was used to perform dynamic simulations due to its high convergence rate and solution accuracy. Due to extreme nonlinearity of the structural behavior under dynamic loading, element size and time increment for the simulations are refined to achieve stable convergence and accurate solutions [13,14].

### 2.1. Analytical Procedure

**Figure 1:**
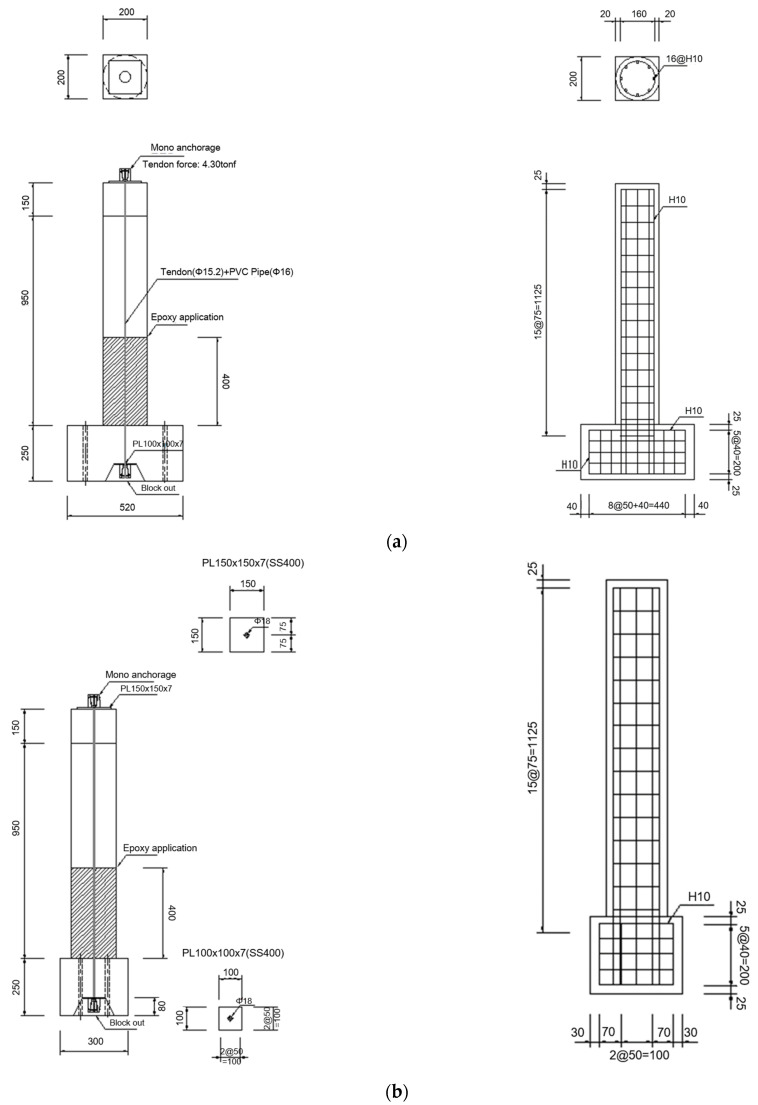
Details of circular cross-sectional RC column specimens: (**a**) side view; (**b**) front view.

**Figure 2:**
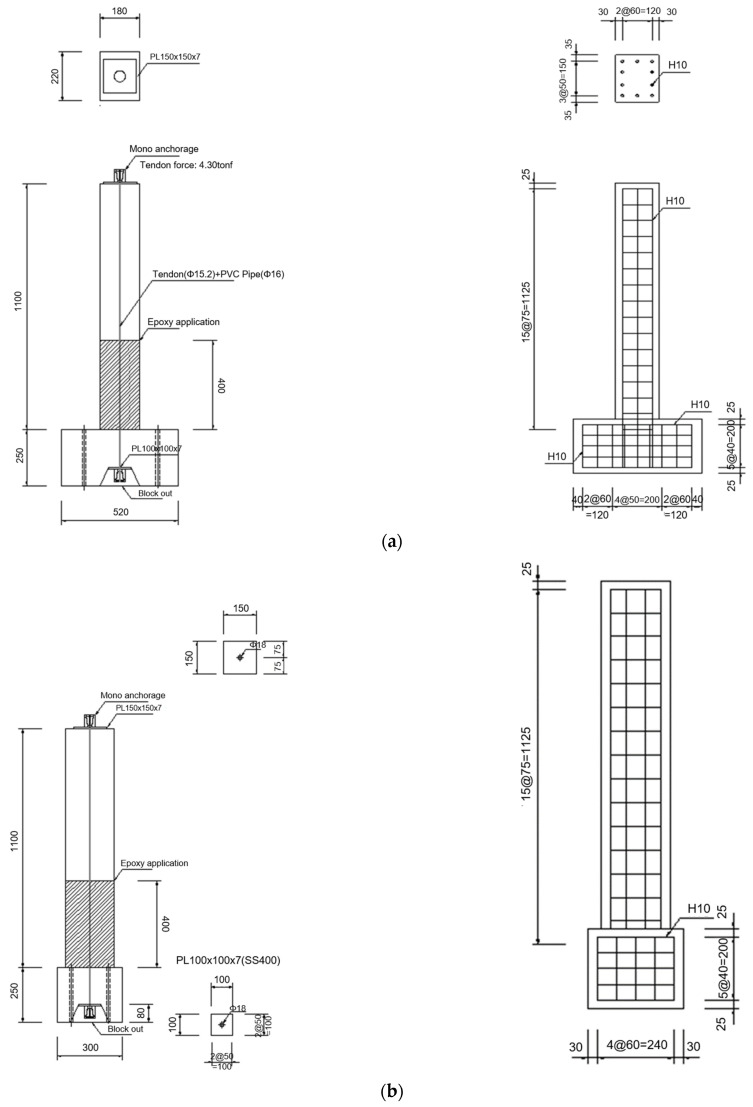
Details of rectangular cross-sectional RC column specimens: (**a**) side view; (**b**) front view.

Figure 1 and Figure 2 present the detailed configuration of the specimens used in the shaking table experiments. The experimental RC columns were designed with a target concrete compressive strength of 30 MPa. For the circular cross-sectional specimens, the column had a diameter of 200 mm and a height of 1025 mm (excluding the basement block), and was reinforced with eight longitudinal D10 rebars and D10 hoop ties spaced at 75 mm intervals. Based on the target reinforcement ratio of 0.0161, the actual reinforcement ratio was constructed as 0.0182. In the case of the rectangular cross-sectional specimens, the column dimensions were 180 mm in width, 220 mm in depth, and 1025 mm in height (excluding the basement block). These specimens were reinforced with ten longitudinal D10 rebars and D10 hoop ties spaced at 75 mm intervals, resulting in a reinforcement ratio of 0.0180.

The material properties, specimen dimensions, and test setup conditions used in the dynamic simulations are as same as those from the experiments. Same as the test specimens, the prestressing force applied to the column is modeled by implementing an expansion coefficient of 1 × 10^−5^ followed by a prestressing force of 3.5 tons (e.g., using a pre-defined field option) to the truss element placed in the center of the column in the longitudinal direction before the application of seismic loading. After the prestressing condition is implemented, the earthquake load of El Centro ground acceleration as a function of time is applied.

**Figure 3:**
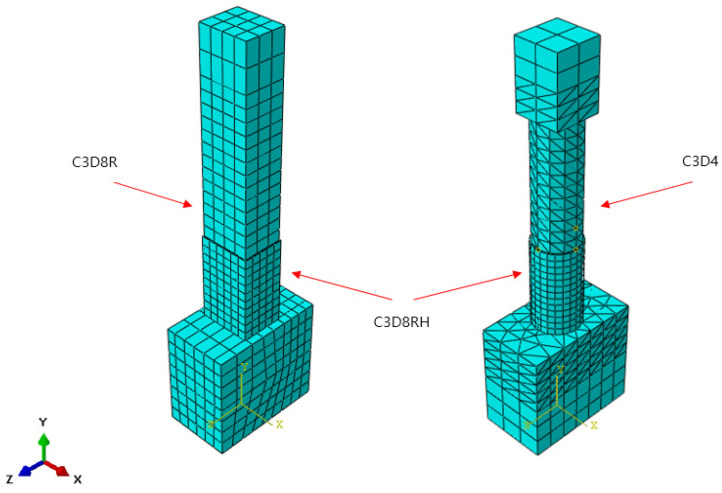
Finite-element meshing.

**Figure 4:**
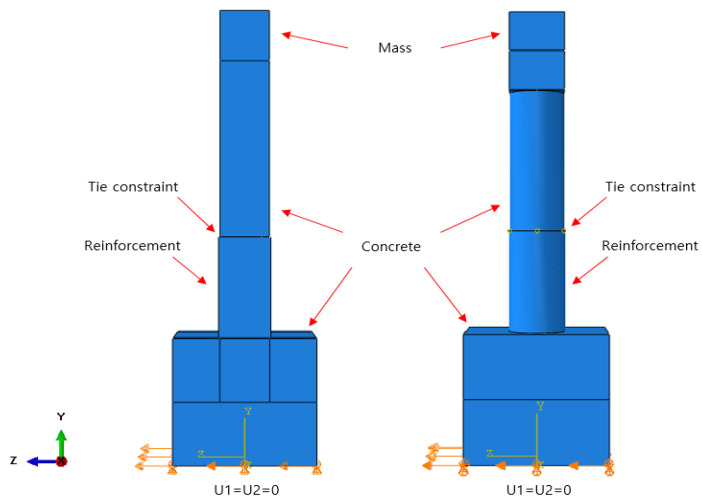
Loading and boundary condition.

For the element type, an incompressible and hyperelastic model of the C3D8RH element of a 3D, eight-node brick type with reduced integration and hourglass control is used, which is given in the ABAQUS Standard library [15,16]. For the GFRP-strengthening plate, an element type S4R (4-node shell element) with reduced integration and hourglass control is used, which is also provided in the library. In order to eliminate shear-locking behavior from occurring during the seismic simulations, a 3D quadrilateral element with 8 nodes (C3D8R) and 4 nodes (C3D4) are applied together. Figure 3 and Figure 4 show the rectangular and circular cross-section column models and corresponding boundary conditions, respectively, used for the dynamic simulations.

In dynamic finite-element analysis, the mesh size plays a critical role in ensuring accurate wave propagation and capturing nonlinear structural behavior. To satisfy the resolution criteria for transient dynamic simulations, the element size was selected to be less than 1/10 of the shortest expected wavelength associated with seismic loading, thereby minimizing numerical dispersion and filtering effects [17]. Regions expected to experience high-stress concentrations, such as the column base and the interface between materials, were further refined to ensure convergence and capture local damage.

For the interaction between different materials—concrete, STPU, and GFRP—tie constraints were implemented in ABAQUS to ensure full displacement compatibility and accurate stress transfer across interfaces. When mesh densities differed between adjacent materials, local mesh refinement was applied to align element edges and maintain geometric compatibility. This strategy enabled the accurate simulation of interface behavior without introducing artificial stress concentrations or numerical instability. A convergence study was performed to verify that the chosen mesh density was sufficient for capturing the global and local response of the system under seismic excitation.

### 2.2. Material Models

#### 2.2.1. Concrete Damage Plasticity

The concrete constitutive model used for the simulation was Concrete Damage Plasticity (CDP). The concrete material was modeled using the Concrete Damage Plasticity (CDP) model available in ABAQUS. This model is based on the yield surface proposed by Lubliner et al., (1989), later modified by Lee and Fenves (1998), and includes both tensile cracking and compressive crushing mechanisms [18,19].

The Concrete Damage Plasticity (CDP) model was initially proposed to represent the inelastic behavior of concrete under monotonic loading and was later enhanced to account for cyclic and dynamic effects. In this model, the yield surface deviates from the classical circular shape and is defined by a shape parameter $K_{c}$, which controls the relative magnitude of tensile and compressive stresses under the same hydrostatic pressure. The value of $K_{c}$ typically ranges from 0.5 to 1.0, with the default being 2/3, which aligns with the Drucker–Prager criterion for concrete.

Additionally, the ratio of biaxial to uniaxial compressive strength ($\sigma_{b0}/\sigma_{c0}$) is another essential parameter for defining the yield surface in CDP. A common default value used in simulations is 1.16, derived from Kupfer’s experimental observations. As concrete is a granular material, it undergoes volumetric expansion during shear-dominated plastic deformation, which must be captured accurately in the flow potential function [20]. The plastic flow of concrete in the CDP framework is governed by two key parameters: the dilation angle (ψ) and the eccentricity (ϵ). The dilation angle typically varies between 5° and 42° for conventional concrete, while the eccentricity is commonly set to 0.1 [13,21]. These parameters significantly influence the concrete’s ability to undergo volumetric plastic flow and affect the simulation’s sensitivity, particularly in relation to confinement effects and reinforcement ratios.

The parameters that need to be defined in the CDP material model are tabulated in Table 1. In the CDP model, the damage state of the element cannot be discretely shown at the integration points. Therefore, the damage state or crack pattern of the simulation results are shown by using equivalent plastic strain (PEEQ) obtained as output. The PEEQ results represent the plastic strains occurring in the element after exceeding the tensile strength of the material. Therefore, when PEEQ results give a positive value, this indicates that the crack has formed in the element [16,22].

#### 2.2.2. PU and GFRP

In order to compare the simulation results of behavior of the RC column strengthened by PU to the experimental results, the Arruda–Boyce model was used as a constitutive model for PU. In the Arruda–Boyce model, hyperelastic material behavior was shown in rubber-like incompressible plastic material. The Arruda-Boyce model uses strain-energy potential as a function of strain to obtain its stress–strain relation, as shown in Equation (1) [23].

(1) $$U=\mu \left\{\frac{1}{2}\left(\bar{I_{1}}-3\right)+\frac{1}{20\lambda_{m}^{2}}\left({\bar{I_{1}}}^{2}-9\right)+\frac{11}{1050\lambda_{m}^{4}}\left({\bar{I_{1}}}^{3}-27\right)+\frac{19}{7000\lambda_{m}^{6}}\left({\bar{I_{1}}}^{4}-81\right)+\frac{519}{673750\lambda_{m}^{8}}\left({\bar{I_{1}}}^{5}-243\right)\right\}+\frac{1}{D}\left(\frac{J_{el}^{2}-1}{2}-\ln J_{el}\right)$$

where $\lambda_{m}$ is the elongation rate in longitudinal direction; $\bar{I}$ is the deviatoric strain invariant; $J_{el}$ is the elastic volumetric rate; and μ and D are the volume compression controlling material coefficients.

In order to simulate the mechanical behavior of STPU, the Arruda–Boyce model was adopted as the constitutive model. Polyurea, being a polymer with a chain-like molecular structure, is capable of undergoing large deformations with strain rates exceeding 100%, and exhibits nonlinear hyperelastic behavior similar to rubber-like materials. Therefore, the Arruda–Boyce model, which is a physically motivated model based on the microstructural mechanics of polymer chains, is considered suitable for capturing such characteristics. Previous studies have also demonstrated its applicability and accuracy in modeling the behavior of polyurea under dynamic loading conditions [24,25].

**Figure 5:**
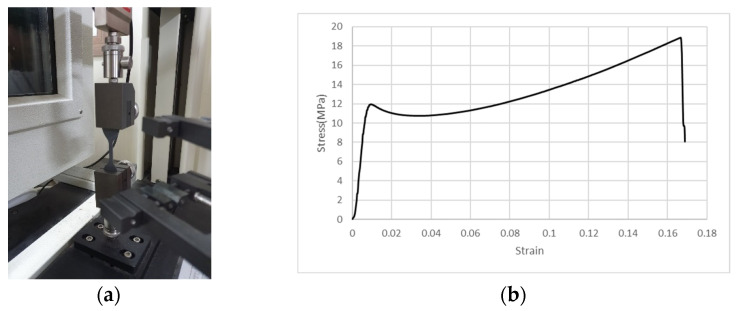
Material evaluation of PU: (**a**) uniaxial tensile test of polyurea; (**b**) stress–strain curve to evaluate hyperelastic material.

In order to obtain the material coefficients to be used in the simulation, a uniaxial tensile test is performed and the stress–strain relation curve is obtained as shown in Figure 5a and Figure 5b, respectively. The tensile strength and percent elongation of PU is 19 MPa and 165%, respectively.

Generally, GFRP rebars and sheets with in-plain debonding failure was modeled using the Hashin damage model [26]. The Hashin damage model was selected for GFRP as it can accurately capture distinct failure modes such as fiber tension, fiber compression, and matrix cracking. In Gliszczynski’s study [27] have demonstrated that the Hashin model is effective in simulating impact-related damage in GFRP plates, showing good agreement with the experimental results. In this model, the stress states based on damage criteria for four damage mechanisms are used, as shown in Equations (2)–(5).

(2) $$F_{f}^{c}={\left(\frac{\bar{\sigma_{11}}}{X_{C}}\right)}^{2}=1,\left(\sigma_{11}<0\right)$$

(3) $$F_{m}^{t}={\left(\frac{\bar{\sigma_{22}}}{Y_{T}}\right)}^{2}+{\left(\frac{\bar{\tau_{12}}}{S_{L}}\right)}^{2}=1,(\sigma_{22}>0)$$

(4) $$F_{f}^{t}={\left(\frac{\bar{\sigma_{11}}}{X_{T}}\right)}^{2}+\alpha {\left(\frac{\bar{\tau_{12}}}{S_{L}}\right)}^{2}=1,\left(\sigma_{11}\geq 0\right)$$

(5) $$F_{m}^{c}={\left(\frac{\bar{\sigma_{22}}}{2S_{T}}\right)}^{2}+\left[{\left(\frac{Y_{C}}{2S_{T}}\right)}^{2}-1\right]\frac{\bar{\sigma_{22}}}{Y_{C}}+{\left(\frac{\bar{\tau_{12}}}{S_{L}}\right)}^{2}=1,\left(\sigma_{22}<0\right)$$

where $F_{f}^{c}$, $F_{m}^{t}$, $F_{f}^{t}$, and $F_{m}^{c}$ are damage criteria of fiber tension, fiber compression, matrix tension, and compression, respectively. $X_{T}$, $X_{C}$, $Y_{T}$, $Y_{C}$, $S_{L}$, and $S_{T}$ are FRP’s longitudinal tensile strength, longitudinal compressive strength, transverse tensile strength, transverse compressive strength, longitudinal shear strength, and transverse shear strength, respectively; the stress tensors of $\sigma_{11}$, $\sigma_{22}$, $\tau_{12}$ represent effective stresses.

## 3. Verification of Numerical Analysis Results

### 3.1. Model Validation

**Figure 6:**
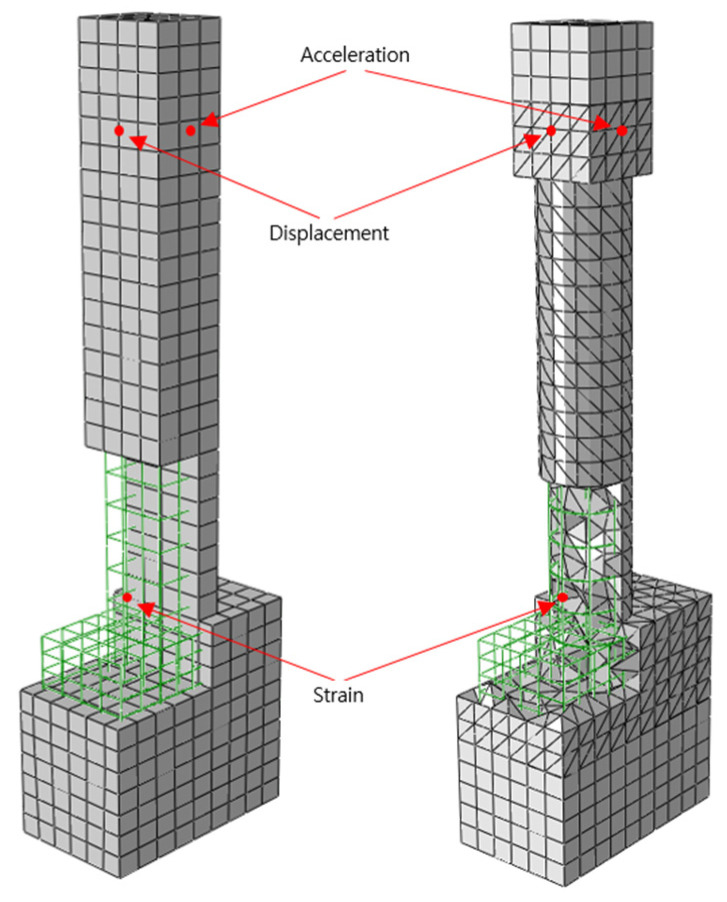
Comparison point for experimental and numerical results.

Based on the experimental results, the simulations are performed. The objective of the simulation work is to quantify the strengthening effect of the application of PU on RC columns under seismic loading. For the calibration of the simulation model, the simulation and experimental results of maximum and minimum deflection, acceleration, and maximum and minimum strain in longitudinal rebars are compared. As shown in Figure 6, the locations where the acceleration and rebar-strain results obtained for both the experiments and simulations are identical.

**Figure 7:**
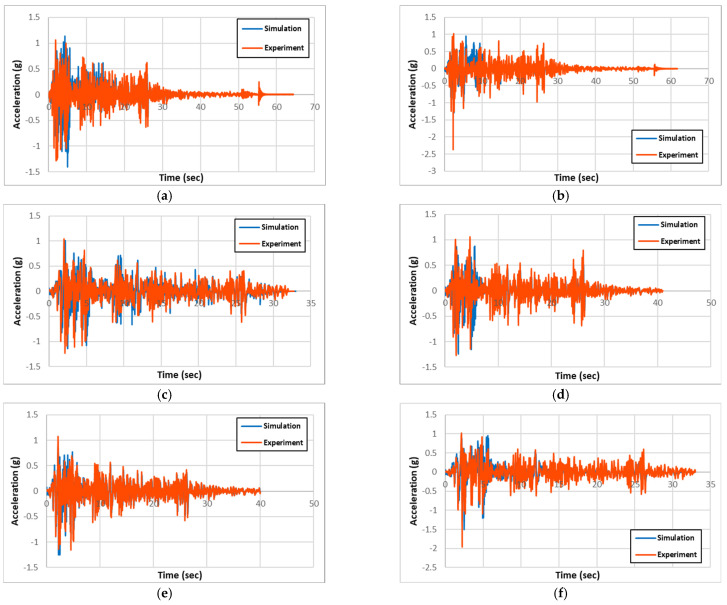
Comparison of experimental and simulation acceleration: (**a**) R-RC; (**b**) C-RC; (**c**) R-PU; (**d**) C-PU; (**e**) R-GFPU; (**f**) C-GFPU. RC: non-strengthened, C-: circular column, R-: rectangular column.

**Figure 8:**
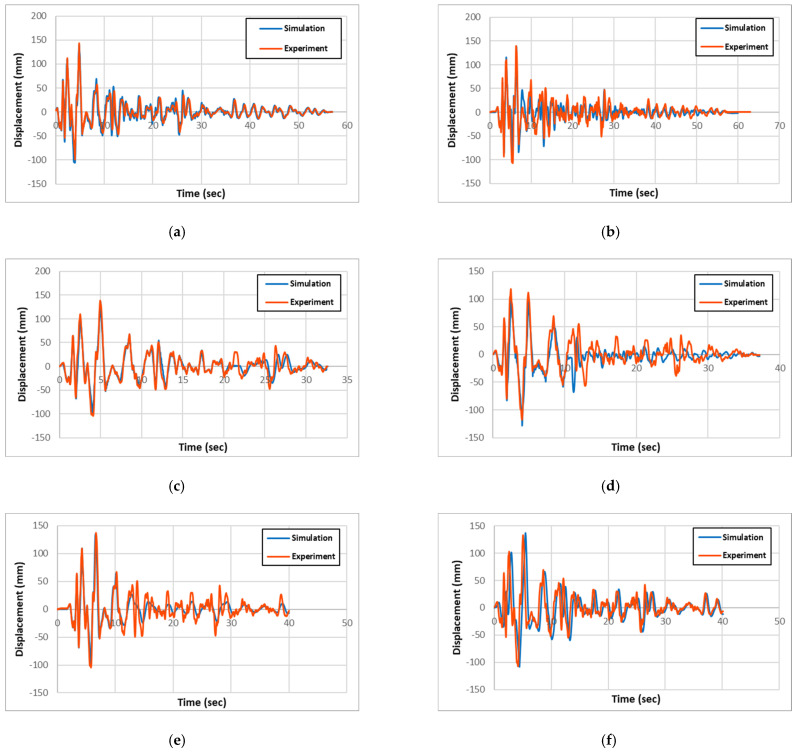
Comparison of experimental and simulation displacement: (**a**) R-RC; (**b**) C-RC; (**c**) R-PU; (**d**) C-PU; (**e**) R-GFPU; (**f**) C-GFPU. RC: non-strengthened, C-: circular column, R-: rectangular column.

**Figure 9:**
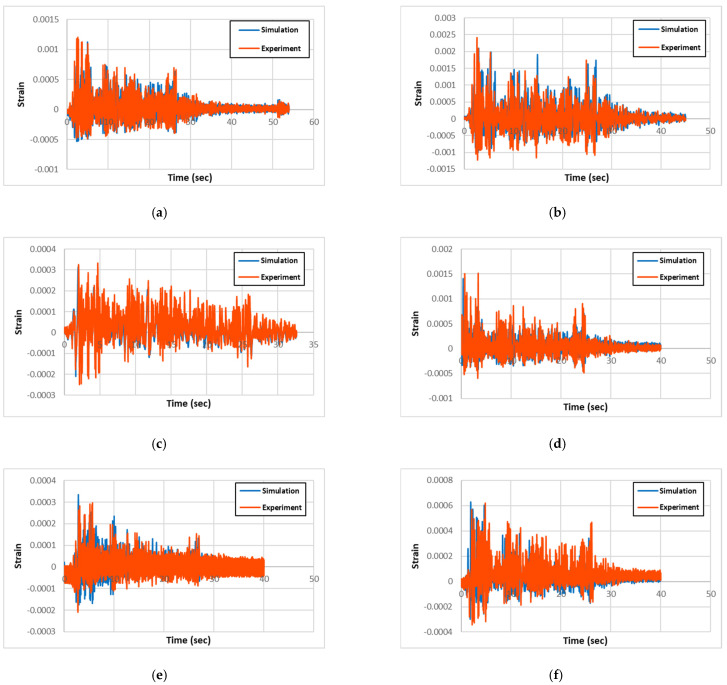
Comparison of experimental and simulation strain: (**a**) R-RC; (**b**) C-RC; (**c**) R-PU; (**d**) C-PU; (**e**) R-GFPU; (**f**) C-GFPU. RC: non-strengthened, C-: circular column, R-: rectangular column.

The experimental and numerical results for acceleration, displacement, and longitudinal-rebar-strain history for all specimen types are presented and compared in Figure 7, Figure 8, and Figure 9, respectively. Also, the maximum column-head displacements and longitudinal rebar strains from the experimental and simulation results for all of the specimen types are summarized in Table 2 and Table 3, respectively. Validation was performed based on maximum column-head displacement and longitudinal rebar strain, which showed good agreement with experimental results. From the comparison of the experimental and simulation results, two significant conclusions can be drawn. One is that the experimental and simulation displacement and strain results are similar within 3% and 14%, respectively, validating the precision of the simulation model.

From the result comparison, the column-head displacements between circular and rectangular columns from both experiments and simulation are relatively the same. Also, the comparison between the non-strengthened RC columns and the strengthened PU and GFPU columns showed that non-strengthened ones had slightly larger displacements in both circular and rectangular columns.

However, the longitudinal-rebar-strain results show that the circular columns have approximately two folds of strains than the rectangular columns from both the experiments and simulations. This trend can be attributed to the fact that the circular columns have much better seismic-resisting capacity than the rectangular columns due to having much larger effective stress-resisting cross-section area than the rectangular ones. When the stains of the circular columns are compared, the non-strengthened RC had approximately 2100~2400 με, the PU-only strengthened one had approximately 1400~1600 με, and the PU- and GFRP-strengthened one had approximately 600 με. This trend in results is logical, since the GFPU strengthening is more effective than the PU-only strengthening, as GFRP applies more confinement and stiffness to the core RC columns, whereas PU only applies conditioning. In the rectangular columns, the non-strengthened RC showed approximately four times more strain than the strengthened ones, caused by large crack damages along the column compared to the concentrated damage at the base joint region in PU- and GFPU-strengthened columns, which are reflected by the strain results in Table 3.

### 3.2. Stiffness Analysis

**Figure 10:**
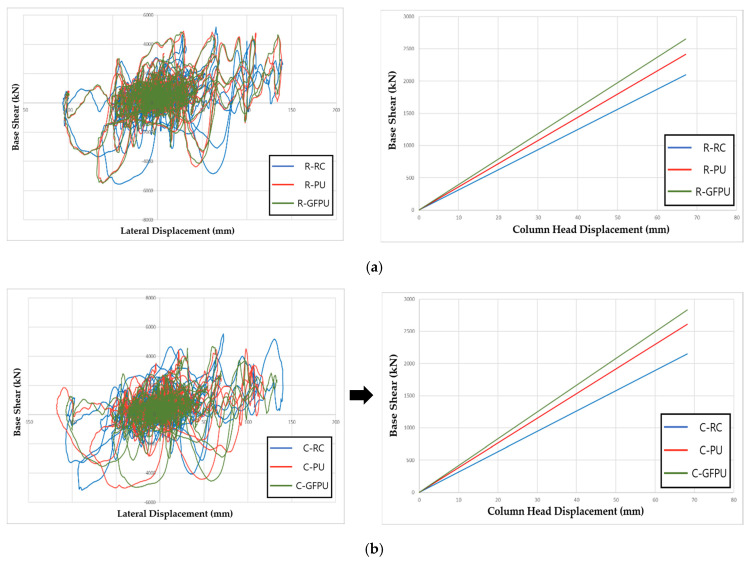
Force–displacement response and comparison of stiffness: (**a**) rectangular cross-section specimen; (**b**) circular cross-section specimen.

To quantitatively analyze the reinforcement effect through the previously constructed analytical model, the lateral shear force–displacement relationship of specimens reinforced with PU and GFRP was compared. In order to increase the effectiveness of the reinforcement-effect analysis, the criteria were set through the PEEQ parameter, which is the failure criteria of the concrete material model that occurred at the 450 mm (the halfway point of the reinforcement position) from the bottom of the test specimen. The stiffness of the structure was analyzed by the ratio of shear force for each specimen based on the later displacement when concrete failure first occurred in the reinforced RC test specimen. As shown in the following Figure 10, both rectangular cross-section specimens and circular cross-section specimens showed a trend of increasing load on the structure compared to the same displacement in the order of GFPU > PU > RC. The stiffness was obtained from the slope of the lateral load–displacement curve at the onset of concrete damage (defined by an initial rise in the PEEQ value at 450 mm in height). As a result of comparing the stiffness of the structure through slope, improvements of 15.04 and 24.46% compared to the non-reinforced specimen were confirmed in R-RC 31.26, R-PU 35.96, and R-GFPU 41.54, respectively, and improvements of 21.35 and 31.75% were confirmed in C-RC 31.53, C-PU 38.26, and C-GFPU 41.54, respectively.

### 3.3. Energy Dissipation

ABAQUS/Standard can show the progress of structural failure and deformation as dissipated energy (ALLDMD) through the structural damage criteria of the properties input in the analysis model. In general, if a structure has excellent rigidity and seismic performance, this increases the energy that can be dissipated as the load action increases, resulting in the high stability of the structure. Notably, the corresponding parameter has a proportional relationship to the failed material volume of the member when the same load is applied [28]. The damage dissipated energy (ALLDMD, M_D) can be calculated using Equation (6).

(6) $$M_{D}=\int_{0}^{T}\int_{V}\sigma \varepsilon^{ck}dVdt$$

In the formula, T represents the duration of ground motion, V is the material volume, σ is the stress, and $\varepsilon^{ck}$ is the cracking strain.

**Figure 11:**
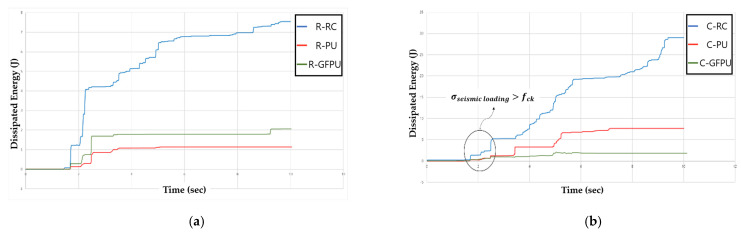
ALLDMD for column specimen using reinforcement method as the variable: (**a**) rectangular cross-section; (**b**) circular cross-section.

Figure 11 presents the comparison of the ALLDMD graphs for all specimens, with data up to the initial 10 s considered to show the macroscopic differences. According to the data, the energy dissipated when the specimen was destroyed was compared for different reinforcement methods. As shown in Figure 11, critical failure occurred in the specimen between 2 and 6 s when the maximum seismic load was applied. Since the failure of the specimen against compression and tensile stress occurs repeatedly due to the characteristics of the seismic loading, it is necessary to analyze the reinforcing effect of the reinforcing material based on a specific period. To analyze the initial loss of the stiffness of the specimen, the dissipated energy of the specimen according to each cross-sectional shape was compared based on the time of initial concrete destruction. Table 4 lists the range of dissipated energy values with this period. Compared to the non-reinforced circular column, the circular cross-section specimen showed a reduction with the range of dissipated energy values of 76% and 82% when PU and GFPU reinforcement were applied, respectively. Compared to the non-reinforced rectangular column, the rectangular cross-section specimen showed a reduction with the range of dissipated energy values of 80% and 60% when PU and GFPU reinforcement were applied, respectively. This occurs because both the PU and GFPU reinforcement cause damage due to the concentration of stress concentration following repeated loads, as the PU was torn off from the corner without a single movement of the PU and GFRP sheets. For a circular cross-sectional specimen without edges, this occurs because the circular cross-sectional specimen has a superior energy dissipation ability than the prismatic cross-sectional specimen due to the formation of a discontinuous surface in the synthesis of PU.

### 3.4. Concrete and Reinforcement Damage Analysis

**Figure 12:**
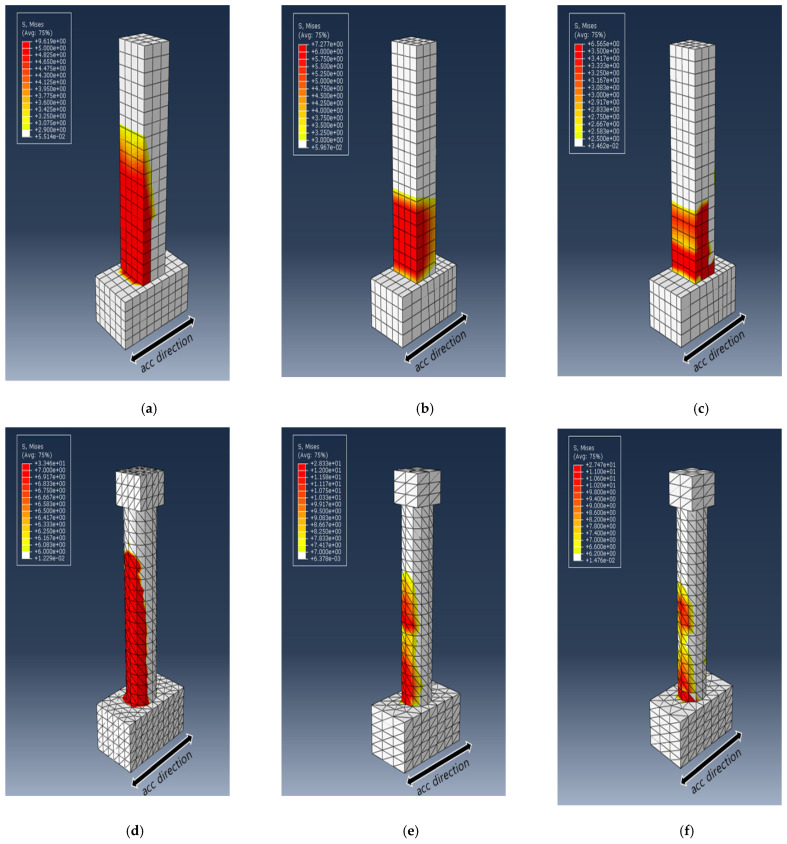
Distribution of von Mises stress (in MPa) for each specimen at the time of initial failure: (**a**) R-RC; (**b**) R-PU; (**c**) R-GFPU; (**d**) C-RC; (**e**) C-PU; (**f**) C-GFPU. RC: non-strengthened, C-: circular column, R-: rectangular column.

**Figure 13:**
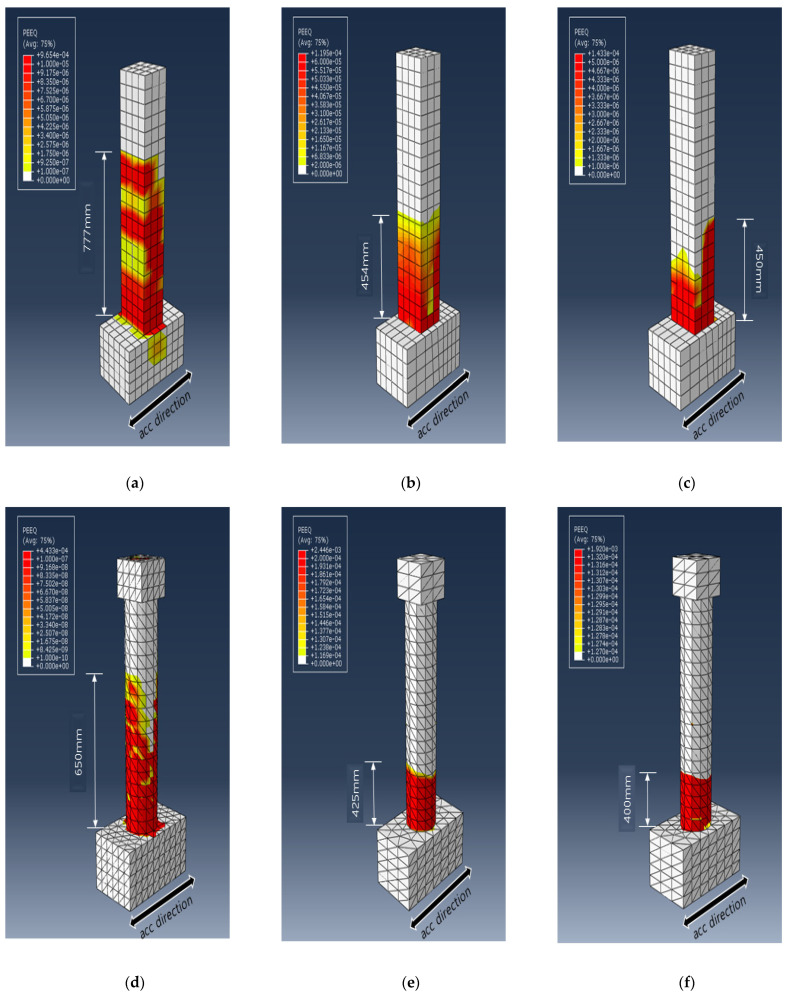
Effective plastic strain (PEEQ) distribution for each specimen at the time of initial failure: (**a**) R-RC; (**b**) R-PU; (**c**) R-GFPU; (**d**) C-RC; (**e**) C-PU; (**f**) C-GFPU. RC: non-strengthened, C-: circular column, R-: rectangular column.

To explain the difference in the trend of the dissipated energy for the different cross-sectional PU and GFPU reinforced specimens, the von Mises stress distribution of each specimen and the degree of concrete fracture were compared at the specific time that the first concrete failure occurred, which has been shown with ALLDMD. PEEQ was used to visualize cracks in the concrete elements, with the CDP model used as the material model of concrete. Figure 12 and Figure 13 show the distribution of the von Mises stress and PEEQ for each specimen. As in the case of the ALLDMD graph, considerable stress occurred in the circular cross-section specimen. In addition, similar to the trend of the dissipated energy, for the square specimens, a wider distribution and larger stress occurred in the GFPU reinforced specimen than those in the PU-reinforced specimen. The contrasting trend was observed for the circular specimen.

The CDP model, used as a concrete material model, is a continuum damage model based on the plastic deformation of concrete. The model assumes tensile cracking and compressive crushing as the main fracture mechanisms of concrete elements. The progression of concrete cracking is controlled by two hardening parameters, which represent tensile and compressive equivalent plastic strains (PEEQ, ε_t_^~pl^, ε_c_^~pl^), both of which are the major failure mechanisms mentioned above. It is affected by tensile and compressive forces. The Concrete Damage Plasticity model lacks the functionality to indicate whether cracks occur at the integration point of the mesh using the material model. Therefore, the concept of effective crack direction can be introduced for the purpose of graphic visualization of crack patterns in concrete specimens. Various criteria have been studied to define the crack direction. In this study, the concept of crack initiation of concrete is used at the integration point where the equivalent plastic strain (PEEQ) is greater than zero, proposed by Lubliner et al. (1989) [18]. Also, the concept assumes that the propagation direction of cracks is parallel to the propagation direction of equivalent plastic strain (PEEQ) [22,29,30]. Through this, the concrete cracks of each specimen were evaluated.

The PEEQ output parameter indicated that the crack occurrence decreased in the order of RC, PU, GFPU when comparing the crack height that progressed in the upper direction of the column from the expected concrete fracture (the foundation and column starting point). It was confirmed that crack heights of 454 mm and 450 mm were generated in the case of R-PU and R-GFPU, respectively, and crack heights of 425 mm and 400 mm were generated in the cases of C-PU and C-GFPU, respectively. As mentioned previously, it can be analyzed that in the case of a rectangular cross-section specimen, discontinuous edges that exist on the surface of the specimen have led to the insufficiency of the reinforcement effect to the reinforcement material compared to the circular cross-sectional test specimen.

Through this approach, it is possible to quantitatively evaluate the degree of structural destruction in the context of the reinforcement effect, and the findings can provide valuable guidance for selecting the optimal reinforcement construction method for actual structures.

## 4. Conclusions

In this paper, numerical models were developed to quantitatively evaluate the seismic reinforcement of STPU-strengthened RC columns on the experimental results of specimens and the numerical results are as follows.

1. In order to implement STPU and GFRP on the FE model, the material model provided by the ABAQUS material library was analyzed. The Arruda–Boyce model was applied to STPU and the Hashin damage model was applied to GFRP.
2. The validity of the analysis model was verified through the main result values derived from RC column specimens. The FE simulation results showed good agreement with experimental measurements, with displacement and strain differences within 3% and 14%, respectively. As a result, it was confirmed that similar trends and data between the experimental results and the analysis results were derived, increasing reliability.
3. As a result of FE modeling, developed based on experimental data, the range of dissipated energy (ALLDMD) and equivalent plastic strains (PEEQ) of the specimen generated were compared. Compared to the non-reinforced circular column, the circular cross-section specimen showed a reduction with the range of dissipated energy values of 63% and 91% when PU and GFPU reinforcement were applied, respectively. Compared to the non-reinforced rectangular column, the rectangular cross-section specimen showed a reduction with the range of dissipated energy values of 82% and 73% when PU and GFPU reinforcement were applied, respectively. The PEEQ output parameter indicated that the crack occurrence decreased in the order of RC, PU-, and GFPU-reinforced specimens when comparing the crack height that progressed in the upper direction of the column.
4. In the case of circular specimens, the performance of GFPU reinforcement was superior to that of PU reinforcement. In the case of the rectangular specimens, the effect of the two reinforcement types was comparable, albeit superior to that of non-reinforced specimens. Compared to the circular cross-sectional specimens, it can be observed that in the case of a rectangular cross-section specimen, discontinuous edges that exist on the surface of the specimen have led to the insufficiency of the reinforcement effect to the reinforcement material. Future studies will explore large-scale applications of STPU and incorporate advanced damage modeling techniques under stochastic seismic loading.

## Figures and Tables

**Table 1:** Material properties for Concrete Damage Plasticity.

Density (kg/m^3^)	Elastic Modulus (MPa)	ν	ψ	ϵ	$\sigma_{b0}/\sigma_{c0}$	$K_{c}$	μ
2400	27,537	0.167	15	0.1	1.16	0.667	0.0005

**Table 2:** Maximum displacement results of the column head for all specimen types.

Specimen	Column Head (mm)		Difference Rate (%)
	Experiment	Simulation	
R-RC	140.17	143.39	2.6
R-PU	138.86	135.74	2.3
R-GFPU	137.41	135.06	1.7
C-RC	139.97	138.9	0.8
C-PU	118.37	128.26	8.4
C-GFPU	133.14	137.04	2.9

**Table 3:** Maximum strain results of the longitudinal rebar for all specimen types.

Specimen	Longitudinal Rebar Strain		Difference Rate (%)
	Experiment	Simulation	
R-RC	1200	1124	6.3
R-PU	330	315	4.5
R-GFPU	300	335	11.7
C-RC	2420	2098	13.3
C-PU	1630	1407	13.7
C-GFPU	620	630	1.6

**Table 4:** Range of ALLDMD and ratio comparison.

Specimen	Range of Dissipated Energy (J)	Ratio
R-RC	4.21	1.00
R-PU	0.85	0.20
R-GFPU	1.69	0.40
C-RC	5.26	1.00
C-PU	1.23	0.24
C-GFPU	0.97	0.18

RC: non-strengthened, C-: circular column, R-: rectangular column.

## Data Availability

The data presented in this research are available on request from the first author due to privacy and confidentiality agreements with the collaborating institution.
